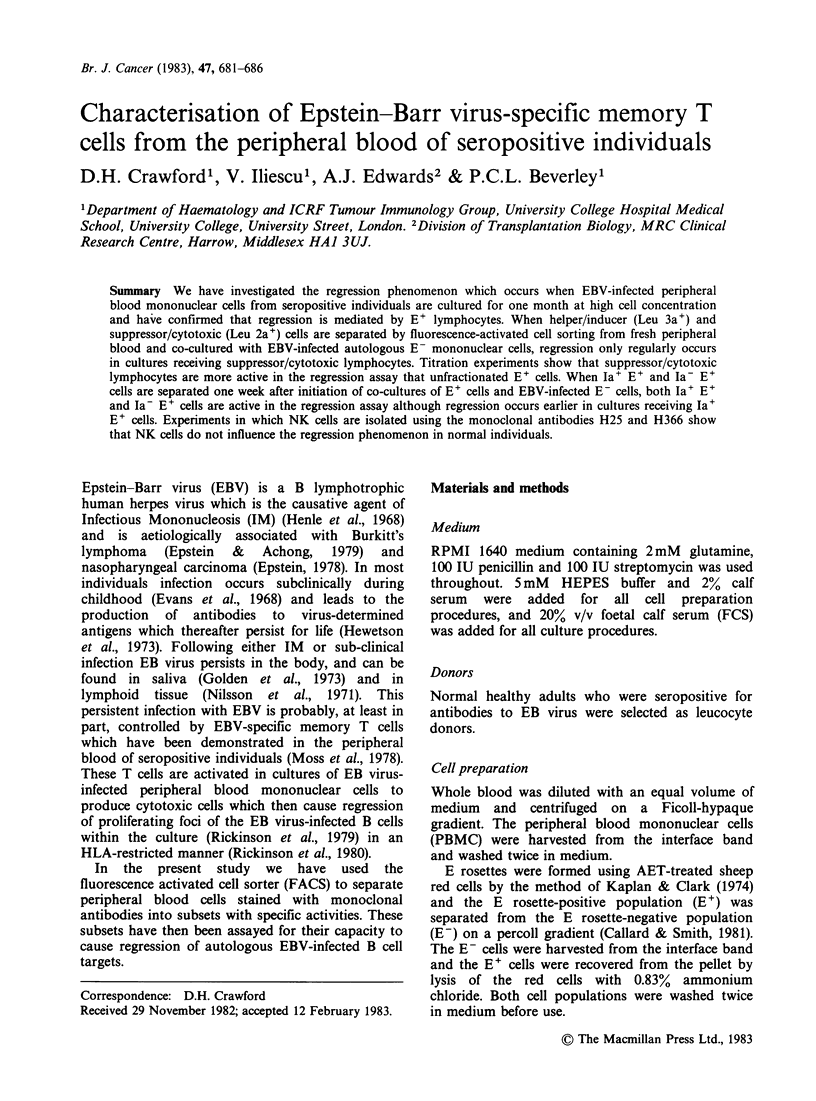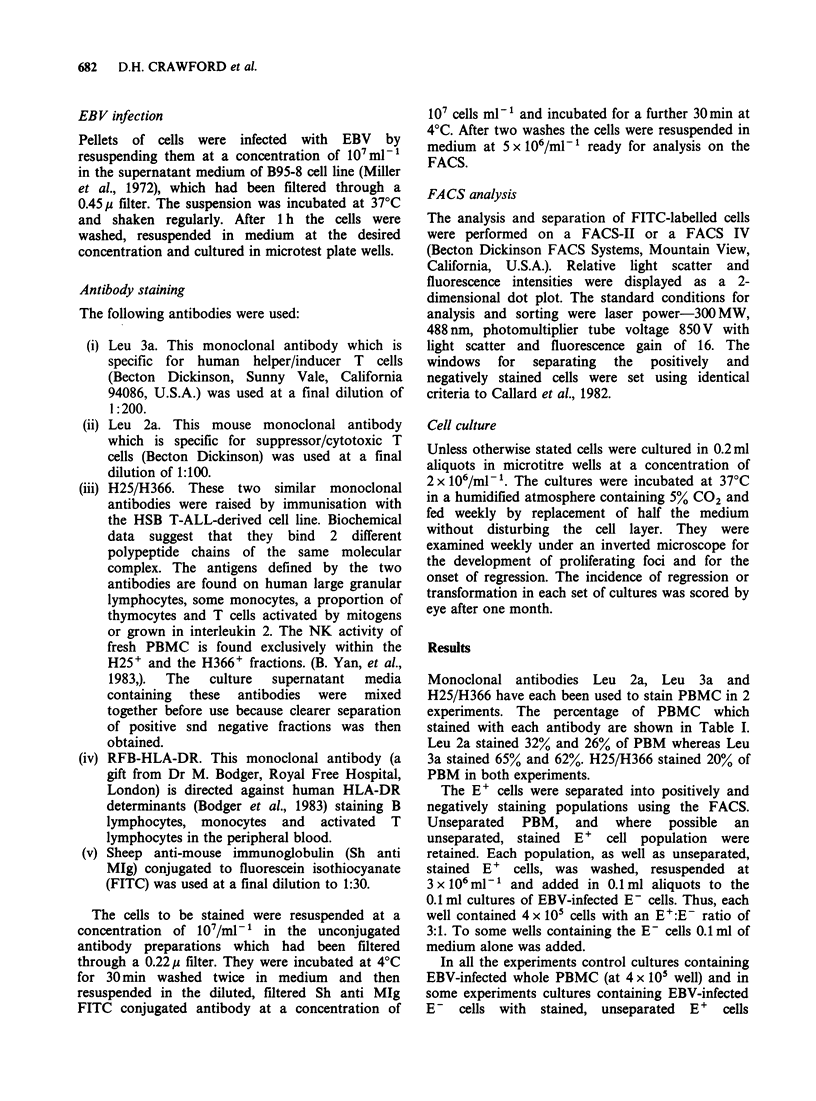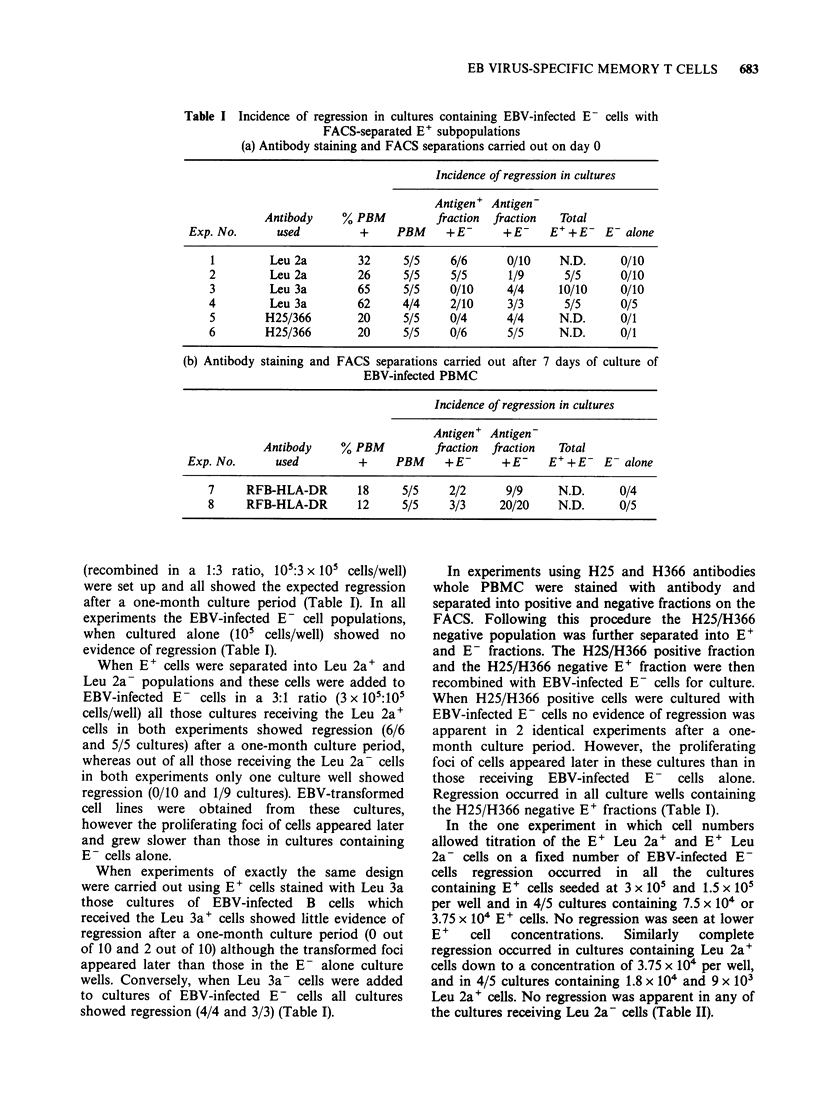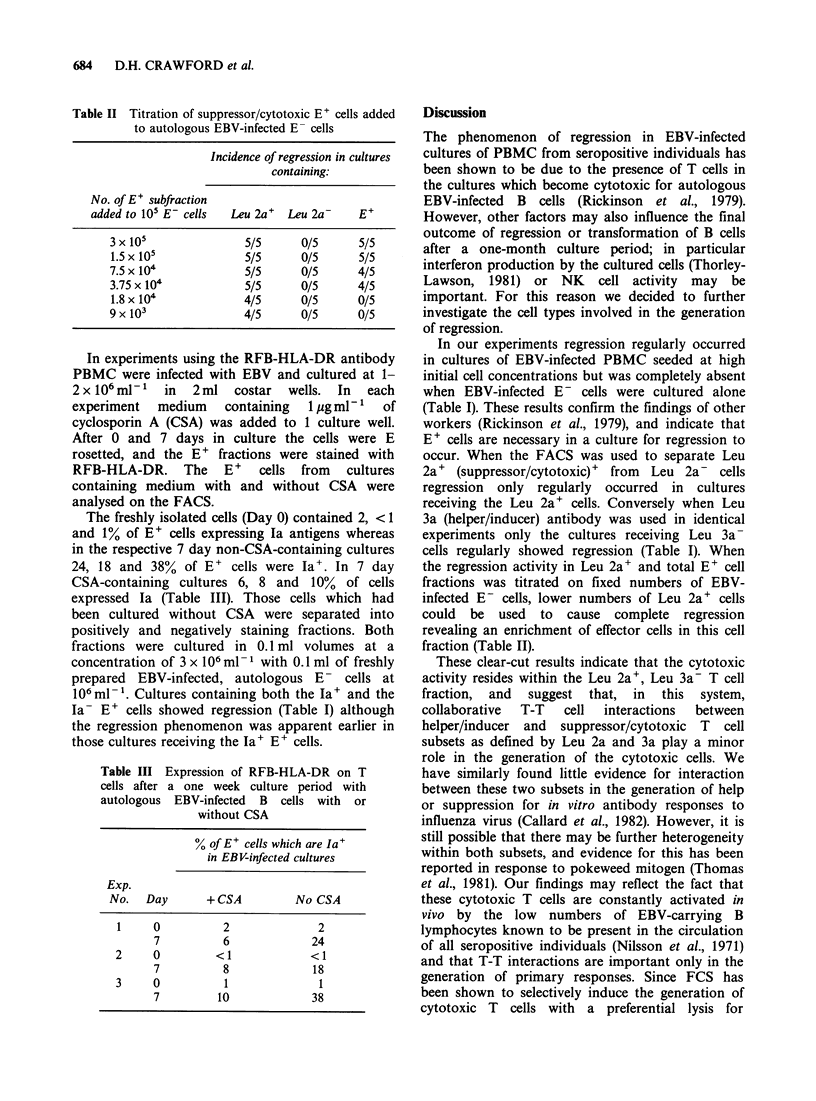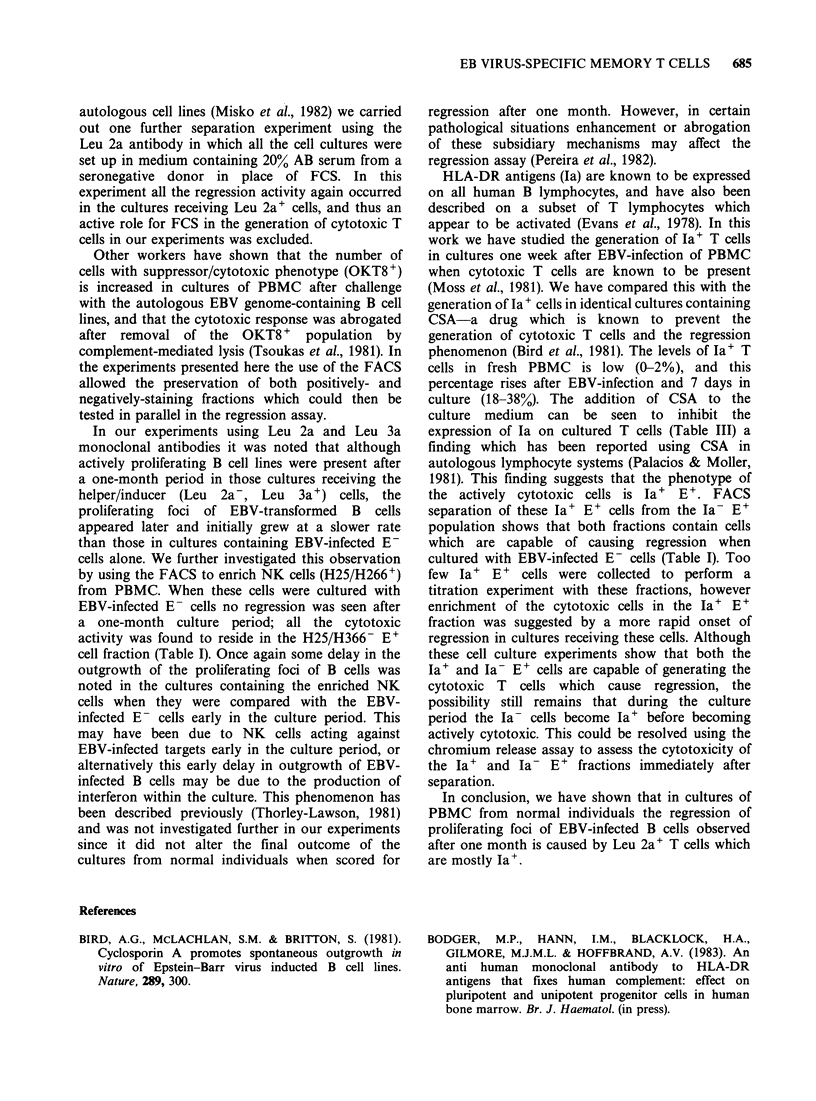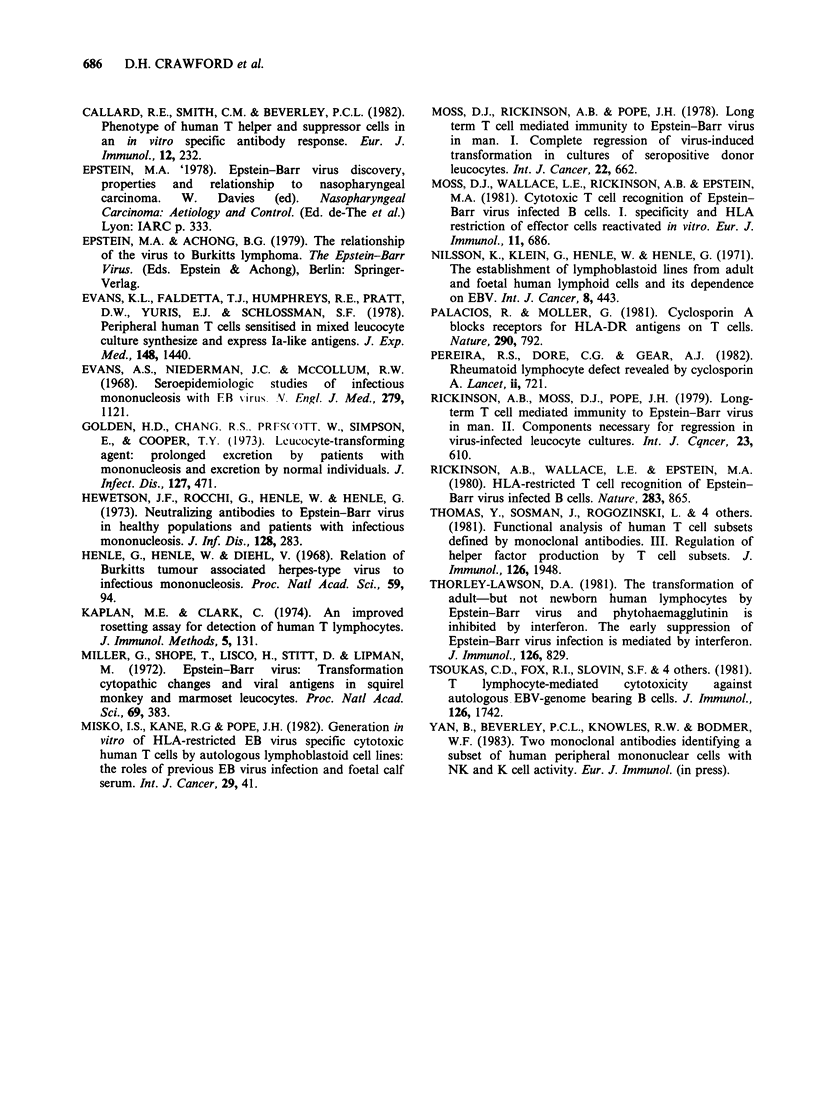# Characterisation of Epstein-Barr virus-specific memory T cells from the peripheral blood of seropositive individuals.

**DOI:** 10.1038/bjc.1983.106

**Published:** 1983-05

**Authors:** D. H. Crawford, V. Iliescu, A. J. Edwards, P. C. Beverley

## Abstract

We have investigated the regression phenomenon which occurs when EBV-infected peripheral blood mononuclear cells from seropositive individuals are cultured for one month at high cell concentration and have confirmed that regression is mediated by E+ lymphocytes. When helper/inducer (Leu 3a+) and suppressor/cytotoxic (Leu 2a+) cells are separated by fluorescence-activated cell sorting from fresh peripheral blood and co-cultured with EBV-infected autologous E- mononuclear cells, regression only regularly occurs in cultures receiving suppressor/cytotoxic lymphocytes. Titration experiments show that suppressor/cytotoxic lymphocytes are more active in the regression assay that unfractionated E+ cells. When Ia+ E+ and Ia- E+ cells are separated one week after initiation of co-cultures of E+ cells and EBV-infected E- cells, both Ia+ E+ and Ia- E+ cells are active in the regression assay although regression occurs earlier in cultures receiving Ia+ E+ cells. Experiments in which NK cells are isolated using the monoclonal antibodies H25 and H366 show that NK cells do not influence the regression phenomenon in normal individuals.


*Br. J. Cancer* (1983), **47**, 681–686

### Characterisation of Epstein–Barr virus-specific memory T cells from the peripheral blood of seropositive individuals

D.H. Crawford[1], V. Iliescu[1], A.J. Edwards[2] & P.C.L. Beverley[1]

[1]*Department of Haematology and ICRF Tumour Immunology Group, University College Hospital Medical School, University College, University Street, London.* [2]*Division of Transplantation Biology, MRC Clinical Research Centre, Harrow, Middlesex HA1 3UJ.*

**Summary** We have investigated the regression phenomenon which occurs when EBV-infected peripheral blood mononuclear cells from seropositive individuals are cultured for one month at high cell concentration and have confirmed that regression is mediated by $E^+$ lymphocytes. When helper/inducer (Leu $3a^+$) and suppressor/cytotoxic (Leu $2a^+$) cells are separated by fluorescence-activated cell sorting from fresh peripheral blood and co-cultured with EBV-infected autologous $E^-$ mononuclear cells, regression only regularly occurs in cultures receiving suppressor/cytotoxic lymphocytes. Titration experiments show that suppressor/cytotoxic lymphocytes are more active in the regression assay that unfractionated $E^+$ cells. When $Ia^+$ $E^+$ and $Ia^-$ $E^+$ cells are separated one week after initiation of co-cultures of $E^+$ cells and EBV-infected $E^-$ cells, both $Ia^+$ $E^+$ and $Ia^-$ $E^+$ cells are active in the regression assay although regression occurs earlier in cultures receiving $Ia^+$ $E^+$ cells. Experiments in which NK cells are isolated using the monoclonal antibodies H25 and H366 show that NK cells do not influence the regression phenomenon in normal individuals.

Epstein–Barr virus (EBV) is a B lymphotrophic human herpes virus which is the causative agent of Infectious Mononucleosis (IM) (Henle *et al.*, 1968) and is aetiologically associated with Burkitt's lymphoma (Epstein & Achong, 1979) and nasopharyngeal carcinoma (Epstein, 1978). In most individuals infection occurs subclinically during childhood (Evans *et al.*, 1968) and leads to the production of antibodies to virus-determined antigens which thereafter persist for life (Hewetson *et al.*, 1973). Following either IM or sub-clinical infection EB virus persists in the body, and can be found in saliva (Golden *et al.*, 1973) and in lymphoid tissue (Nilsson *et al.*, 1971). This persistent infection with EBV is probably, at least in part, controlled by EBV-specific memory T cells which have been demonstrated in the peripheral blood of seropositive individuals (Moss *et al.*, 1978). These T cells are activated in cultures of EB virus-infected peripheral blood mononuclear cells to produce cytotoxic cells which then cause regression of proliferating foci of the EB virus-infected B cells within the culture (Rickinson *et al.*, 1979) in an HLA-restricted manner (Rickinson *et al.*, 1980).

In the present study we have used the fluorescence activated cell sorter (FACS) to separate peripheral blood cells stained with monoclonal antibodies into subsets with specific activities. These subsets have then been assayed for their capacity to cause regression of autologous EBV-infected B cell targets.

Correspondence: D.H. Crawford
Received 29 November 1982; accepted 12 February 1983.

### Materials and methods

### *Medium*

RPMI 1640 medium containing 2 mM glutamine, 100 IU penicillin and 100 IU streptomycin was used throughout. 5 mM HEPES buffer and 2% calf serum were added for all cell preparation procedures, and 20% v/v foetal calf serum (FCS) was added for all culture procedures.

### *Donors*

Normal healthy adults who were seropositive for antibodies to EB virus were selected as leucocyte donors.

### *Cell preparation*

Whole blood was diluted with an equal volume of medium and centrifuged on a Ficoll-hypaque gradient. The peripheral blood mononuclear cells (PBMC) were harvested from the interface band and washed twice in medium.

E rosettes were formed using AET-treated sheep red cells by the method of Kaplan & Clark (1974) and the E rosette-positive population ($E^+$) was separated from the E rosette-negative population ($E^-$) on a percoll gradient (Callard & Smith, 1981). The $E^-$ cells were harvested from the interface band and the $E^+$ cells were recovered from the pellet by lysis of the red cells with 0.83% ammonium chloride. Both cell populations were washed twice in medium before use.

© The Macmillan Press Ltd., 1983

*EBV infection*

Pellets of cells were infected with EBV by resuspending them at a concentration of $10^7 ml^{-1}$ in the supernatant medium of B95-8 cell line (Miller *et al.*, 1972), which had been filtered through a 0.45 $\mu$ filter. The suspension was incubated at 37°C and shaken regularly. After 1 h the cells were washed, resuspended in medium at the desired concentration and cultured in microtest plate wells.

*Antibody staining*

The following antibodies were used:

(i) Leu 3a. This monoclonal antibody which is specific for human helper/inducer T cells (Becton Dickinson, Sunny Vale, California 94086, U.S.A.) was used at a final dilution of 1:200.
(ii) Leu 2a. This mouse monoclonal antibody which is specific for suppressor/cytotoxic T cells (Becton Dickinson) was used at a final dilution of 1:100.
(iii) H25/H366. These two similar monoclonal antibodies were raised by immunisation with the HSB T-ALL-derived cell line. Biochemical data suggest that they bind 2 different polypeptide chains of the same molecular complex. The antigens defined by the two antibodies are found on human large granular lymphocytes, some monocytes, a proportion of thymocytes and T cells activated by mitogens or grown in interleukin 2. The NK activity of fresh PBMC is found exclusively within the $H25^+$ and the $H366^+$ fractions. (B. Yan, *et al.*, 1983,). The culture supernatant media containing these antibodies were mixed together before use because clearer separation of positive snd negative fractions was then obtained.
(iv) RFB-HLA-DR. This monoclonal antibody (a gift from Dr M. Bodger, Royal Free Hospital, London) is directed against human HLA-DR determinants (Bodger *et al.*, 1983) staining B lymphocytes, monocytes and activated T lymphocytes in the peripheral blood.
(v) Sheep anti-mouse immunoglobulin (Sh anti MIg) conjugated to fluorescein isothiocyanate (FITC) was used at a final dilution to 1:30.

The cells to be stained were resuspended at a concentration of $10^7/ml^{-1}$ in the unconjugated antibody preparations which had been filtered through a 0.22 $\mu$ filter. They were incubated at 4°C for 30 min washed twice in medium and then resuspended in the diluted, filtered Sh anti MIg FITC conjugated antibody at a concentration of $10^7$ cells $ml^{-1}$ and incubated for a further 30 min at 4°C. After two washes the cells were resuspended in medium at $5 \times 10^6/ml^{-1}$ ready for analysis on the FACS.

*FACS analysis*

The analysis and separation of FITC-labelled cells were performed on a FACS-II or a FACS IV (Becton Dickinson FACS Systems, Mountain View, California, U.S.A.). Relative light scatter and fluorescence intensities were displayed as a 2-dimensional dot plot. The standard conditions for analysis and sorting were laser power—300 MW, 488 nm, photomultiplier tube voltage 850 V with light scatter and fluorescence gain of 16. The windows for separating the positively and negatively stained cells were set using identical criteria to Callard *et al.*, 1982.

*Cell culture*

Unless otherwise stated cells were cultured in 0.2 ml aliquots in microtitre wells at a concentration of $2 \times 10^6/ml^{-1}$. The cultures were incubated at 37°C in a humidified atmosphere containing 5% $CO_2$ and fed weekly by replacement of half the medium without disturbing the cell layer. They were examined weekly under an inverted microscope for the development of proliferating foci and for the onset of regression. The incidence of regression or transformation in each set of cultures was scored by eye after one month.

### Results

Monoclonal antibodies Leu 2a, Leu 3a and H25/H366 have each been used to stain PBMC in 2 experiments. The percentage of PBMC which stained with each antibody are shown in Table I. Leu 2a stained 32% and 26% of PBM whereas Leu 3a stained 65% and 62%. H25/H366 stained 20% of PBM in both experiments.

The $E^+$ cells were separated into positively and negatively staining populations using the FACS. Unseparated PBM, and where possible an unseparated, stained $E^+$ cell population were retained. Each population, as well as unseparated, stained $E^+$ cells, was washed, resuspended at $3 \times 10^6 ml^{-1}$ and added in 0.1 ml aliquots to the 0.1 ml cultures of EBV-infected $E^-$ cells. Thus, each well contained $4 \times 10^5$ cells with an $E^+$:$E^-$ ratio of 3:1. To some wells containing the $E^-$ cells 0.1 ml of medium alone was added.

In all the experiments control cultures containing EBV-infected whole PBMC (at $4 \times 10^5$ well) and in some experiments cultures containing EBV-infected $E^-$ cells with stained, unseparated $E^+$ cells

**Table I** Incidence of regression in cultures containing EBV-infected $E^-$ cells with FACS-separated $E^+$ subpopulations

(a) Antibody staining and FACS separations carried out on day 0

| | | | Incidence of regression in cultures | | | | |
|---|---|---|---|---|---|---|---|
| *Exp. No.* | *Antibody used* | *% PBM +* | *PBM* | *Antigen$^+$ fraction $+E^-$* | *Antigen$^-$ fraction $+E^-$* | *Total $E^+ + E^-$* | *$E^-$ alone* |
| 1 | Leu 2a | 32 | 5/5 | 6/6 | 0/10 | N.D. | 0/10 |
| 2 | Leu 2a | 26 | 5/5 | 5/5 | 1/9 | 5/5 | 0/10 |
| 3 | Leu 3a | 65 | 5/5 | 0/10 | 4/4 | 10/10 | 0/10 |
| 4 | Leu 3a | 62 | 4/4 | 2/10 | 3/3 | 5/5 | 0/5 |
| 5 | H25/366 | 20 | 5/5 | 0/4 | 4/4 | N.D. | 0/1 |
| 6 | H25/366 | 20 | 5/5 | 0/6 | 5/5 | N.D. | 0/1 |

(b) Antibody staining and FACS separations carried out after 7 days of culture of EBV-infected PBMC

| | | | Incidence of regression in cultures | | | | |
|---|---|---|---|---|---|---|---|
| *Exp. No.* | *Antibody used* | *% PBM +* | *PBM* | *Antigen$^+$ fraction $+E^-$* | *Antigen$^-$ fraction $+E^-$* | *Total $E^+ + E^-$* | *$E^-$ alone* |
| 7 | RFB-HLA-DR | 18 | 5/5 | 2/2 | 9/9 | N.D. | 0/4 |
| 8 | RFB-HLA-DR | 12 | 5/5 | 3/3 | 20/20 | N.D. | 0/5 |

(recombined in a 1:3 ratio, $10^5$:$3 \times 10^5$ cells/well) were set up and all showed the expected regression after a one-month culture period (Table I). In all experiments the EBV-infected $E^-$ cell populations, when cultured alone ($10^5$ cells/well) showed no evidence of regression (Table I).

When $E^+$ cells were separated into Leu $2a^+$ and Leu $2a^-$ populations and these cells were added to EBV-infected $E^-$ cells in a 3:1 ratio ($3 \times 10^5$:$10^5$ cells/well) all those cultures receiving the Leu $2a^+$ cells in both experiments showed regression (6/6 and 5/5 cultures) after a one-month culture period, whereas out of all those receiving the Leu $2a^-$ cells in both experiments only one culture well showed regression (0/10 and 1/9 cultures). EBV-transformed cell lines were obtained from these cultures, however the proliferating foci of cells appeared later and grew slower than those in cultures containing $E^-$ cells alone.

When experiments of exactly the same design were carried out using $E^+$ cells stained with Leu 3a those cultures of EBV-infected B cells which received the Leu $3a^+$ cells showed little evidence of regression after a one-month culture period (0 out of 10 and 2 out of 10) although the transformed foci appeared later than those in the $E^-$ alone culture wells. Conversely, when Leu $3a^-$ cells were added to cultures of EBV-infected $E^-$ cells all cultures showed regression (4/4 and 3/3) (Table I).

In experiments using H25 and H366 antibodies whole PBMC were stained with antibody and separated into positive and negative fractions on the FACS. Following this procedure the H25/H366 negative population was further separated into $E^+$ and $E^-$ fractions. The H2S/H366 positive fraction and the H25/H366 negative $E^+$ fraction were then recombined with EBV-infected $E^-$ cells for culture. When H25/H366 positive cells were cultured with EBV-infected $E^-$ cells no evidence of regression was apparent in 2 identical experiments after a one-month culture period. However, the proliferating foci of cells appeared later in these cultures than in those receiving EBV-infected $E^-$ cells alone. Regression occurred in all culture wells containing the H25/H366 negative $E^+$ fractions (Table I).

In the one experiment in which cell numbers allowed titration of the $E^+$ Leu $2a^+$ and $E^+$ Leu $2a^-$ cells on a fixed number of EBV-infected $E^-$ cells regression occurred in all the cultures containing $E^+$ cells seeded at $3 \times 10^5$ and $1.5 \times 10^5$ per well and in 4/5 cultures containing $7.5 \times 10^4$ or $3.75 \times 10^4$ $E^+$ cells. No regression was seen at lower $E^+$ cell concentrations. Similarly complete regression occurred in cultures containing Leu $2a^+$ cells down to a concentration of $3.75 \times 10^4$ per well, and in 4/5 cultures containing $1.8 \times 10^4$ and $9 \times 10^3$ Leu $2a^+$ cells. No regression was apparent in any of the cultures receiving Leu $2a^-$ cells (Table II).

**Table II** Titration of suppressor/cytotoxic $E^+$ cells added to autologous EBV-infected $E^-$ cells

| *No. of $E^+$ subfraction added to $10^5$ $E^-$ cells* | *Incidence of regression in cultures containing:* Leu $2a^+$ | Leu $2a^-$ | $E^+$ |
|---|---|---|---|
| $3\times10^5$ | 5/5 | 0/5 | 5/5 |
| $1.5\times10^5$ | 5/5 | 0/5 | 5/5 |
| $7.5\times10^4$ | 5/5 | 0/5 | 4/5 |
| $3.75\times10^4$ | 5/5 | 0/5 | 4/5 |
| $1.8\times10^4$ | 4/5 | 0/5 | 0/5 |
| $9\times10^3$ | 4/5 | 0/5 | 0/5 |

In experiments using the RFB-HLA-DR antibody PBMC were infected with EBV and cultured at 1–$2\times10^6\,ml^{-1}$ in 2 ml costar wells. In each experiment medium containing $1\,\mu g\,ml^{-1}$ of cyclosporin A (CSA) was added to 1 culture well. After 0 and 7 days in culture the cells were E rosetted, and the $E^+$ fractions were stained with RFB-HLA-DR. The $E^+$ cells from cultures containing medium with and without CSA were analysed on the FACS.

The freshly isolated cells (Day 0) contained 2, <1 and 1% of $E^+$ cells expressing Ia antigens whereas in the respective 7 day non-CSA-containing cultures 24, 18 and 38% of $E^+$ cells were $Ia^+$. In 7 day CSA-containing cultures 6, 8 and 10% of cells expressed Ia (Table III). Those cells which had been cultured without CSA were separated into positively and negatively staining fractions. Both fractions were cultured in 0.1 ml volumes at a concentration of $3\times10^6\,ml^{-1}$ with 0.1 ml of freshly prepared EBV-infected, autologous $E^-$ cells at $10^6\,ml^{-1}$. Cultures containing both the $Ia^+$ and the $Ia^-$ $E^+$ cells showed regression (Table I) although the regression phenomenon was apparent earlier in those cultures receiving the $Ia^+$ $E^+$ cells.

**Table III** Expression of RFB-HLA-DR on T cells after a one week culture period with autologous EBV-infected B cells with or without CSA

| *Exp. No.* | *Day* | *% of $E^+$ cells which are $Ia^+$ in EBV-infected cultures* +CSA | *No CSA* |
|---|---|---|---|
| 1 | 0 | 2 | 2 |
| | 7 | 6 | 24 |
| 2 | 0 | <1 | <1 |
| | 7 | 8 | 18 |
| 3 | 0 | 1 | 1 |
| | 7 | 10 | 38 |

### Discussion

The phenomenon of regression in EBV-infected cultures of PBMC from seropositive individuals has been shown to be due to the presence of T cells in the cultures which become cytotoxic for autologous EBV-infected B cells (Rickinson *et al.*, 1979). However, other factors may also influence the final outcome of regression or transformation of B cells after a one-month culture period; in particular interferon production by the cultured cells (Thorley-Lawson, 1981) or NK cell activity may be important. For this reason we decided to further investigate the cell types involved in the generation of regression.

In our experiments regression regularly occurred in cultures of EBV-infected PBMC seeded at high initial cell concentrations but was completely absent when EBV-infected $E^-$ cells were cultured alone (Table I). These results confirm the findings of other workers (Rickinson *et al.*, 1979), and indicate that $E^+$ cells are necessary in a culture for regression to occur. When the FACS was used to separate Leu $2a^+$ (suppressor/cytotoxic)$^+$ from Leu $2a^-$ cells regression only regularly occurred in cultures receiving the Leu $2a^+$ cells. Conversely when Leu 3a (helper/inducer) antibody was used in identical experiments only the cultures receiving Leu $3a^-$ cells regularly showed regression (Table I). When the regression activity in Leu $2a^+$ and total $E^+$ cell fractions was titrated on fixed numbers of EBV-infected $E^-$ cells, lower numbers of Leu $2a^+$ cells could be used to cause complete regression revealing an enrichment of effector cells in this cell fraction (Table II).

These clear-cut results indicate that the cytotoxic activity resides within the Leu $2a^+$, Leu $3a^-$ T cell fraction, and suggest that, in this system, collaborative T-T cell interactions between helper/inducer and suppressor/cytotoxic T cell subsets as defined by Leu 2a and 3a play a minor role in the generation of the cytotoxic cells. We have similarly found little evidence for interaction between these two subsets in the generation of help or suppression for *in vitro* antibody responses to influenza virus (Callard *et al.*, 1982). However, it is still possible that there may be further heterogeneity within both subsets, and evidence for this has been reported in response to pokeweed mitogen (Thomas *et al.*, 1981). Our findings may reflect the fact that these cytotoxic T cells are constantly activated *in vivo* by the low numbers of EBV-carrying B lymphocytes known to be present in the circulation of all seropositive individuals (Nilsson *et al.*, 1971) and that T-T interactions are important only in the generation of primary responses. Since FCS has been shown to selectively induce the generation of cytotoxic T cells with a preferential lysis for autologous cell lines (Misko *et al.*, 1982) we carried out one further separation experiment using the Leu 2a antibody in which all the cell cultures were set up in medium containing 20% AB serum from a seronegative donor in place of FCS. In this experiment all the regression activity again occurred in the cultures receiving Leu 2a$^+$ cells, and thus an active role for FCS in the generation of cytotoxic T cells in our experiments was excluded.

Other workers have shown that the number of cells with suppressor/cytotoxic phenotype (OKT8$^+$) is increased in cultures of PBMC after challenge with the autologous EBV genome-containing B cell lines, and that the cytotoxic response was abrogated after removal of the OKT8$^+$ population by complement-mediated lysis (Tsoukas *et al.*, 1981). In the experiments presented here the use of the FACS allowed the preservation of both positively- and negatively-staining fractions which could then be tested in parallel in the regression assay.

In our experiments using Leu 2a and Leu 3a monoclonal antibodies it was noted that although actively proliferating B cell lines were present after a one-month period in those cultures receiving the helper/inducer (Leu 2a$^-$, Leu 3a$^+$) cells, the proliferating foci of EBV-transformed B cells appeared later and initially grew at a slower rate than those in cultures containing EBV-infected E$^-$ cells alone. We further investigated this observation by using the FACS to enrich NK cells (H25/H266$^+$) from PBMC. When these cells were cultured with EBV-infected E$^-$ cells no regression was seen after a one-month culture period; all the cytotoxic activity was found to reside in the H25/H366$^-$ E$^+$ cell fraction (Table I). Once again some delay in the outgrowth of the proliferating foci of B cells was noted in the cultures containing the enriched NK cells when they were compared with the EBV-infected E$^-$ cells early in the culture period. This may have been due to NK cells acting against EBV-infected targets early in the culture period, or alternatively this early delay in outgrowth of EBV-infected B cells may be due to the production of interferon within the culture. This phenomenon has been described previously (Thorley-Lawson, 1981) and was not investigated further in our experiments since it did not alter the final outcome of the cultures from normal individuals when scored for regression after one month. However, in certain pathological situations enhancement or abrogation of these subsidiary mechanisms may affect the regression assay (Pereira *et al.*, 1982).

HLA-DR antigens (Ia) are known to be expressed on all human B lymphocytes, and have also been described on a subset of T lymphocytes which appear to be activated (Evans *et al.*, 1978). In this work we have studied the generation of Ia$^+$ T cells in cultures one week after EBV-infection of PBMC when cytotoxic T cells are known to be present (Moss *et al.*, 1981). We have compared this with the generation of Ia$^+$ cells in identical cultures containing CSA—a drug which is known to prevent the generation of cytotoxic T cells and the regression phenomenon (Bird *et al.*, 1981). The levels of Ia$^+$ T cells in fresh PBMC is low (0–2%), and this percentage rises after EBV-infection and 7 days in culture (18–38%). The addition of CSA to the culture medium can be seen to inhibit the expression of Ia on cultured T cells (Table III) a finding which has been reported using CSA in autologous lymphocyte systems (Palacios & Moller, 1981). This finding suggests that the phenotype of the actively cytotoxic cells is Ia$^+$ E$^+$. FACS separation of these Ia$^+$ E$^+$ cells from the Ia$^-$ E$^+$ population shows that both fractions contain cells which are capable of causing regression when cultured with EBV-infected E$^-$ cells (Table I). Too few Ia$^+$ E$^+$ cells were collected to perform a titration experiment with these fractions, however enrichment of the cytotoxic cells in the Ia$^+$ E$^+$ fraction was suggested by a more rapid onset of regression in cultures receiving these cells. Although these cell culture experiments show that both the Ia$^+$ and Ia$^-$ E$^+$ cells are capable of generating the cytotoxic T cells which cause regression, the possibility still remains that during the culture period the Ia$^-$ cells become Ia$^+$ before becoming actively cytotoxic. This could be resolved using the chromium release assay to assess the cytotoxicity of the Ia$^+$ and Ia$^-$ E$^+$ fractions immediately after separation.

In conclusion, we have shown that in cultures of PBMC from normal individuals the regression of proliferating foci of EBV-infected B cells observed after one month is caused by Leu 2a$^+$ T cells which are mostly Ia$^+$.

### References

BIRD, A.G., MCLACHLAN, S.M. & BRITTON, S. (1981). Cyclosporin A promotes spontaneous outgrowth *in vitro* of Epstein–Barr virus inducted B cell lines. *Nature*, **289**, 300.

BODGER, M.P., HANN, I.M., BLACKLOCK, H.A., GILMORE, M.J.M.L. & HOFFBRAND, A.V. (1983). An anti human monoclonal antibody to HLA-DR antigens that fixes human complement: effect on pluripotent and unipotent progenitor cells in human bone marrow. *Br. J. Haematol.* (in press).

CALLARD, R.E., SMITH, C.M. & BEVERLEY, P.C.L. (1982). Phenotype of human T helper and suppressor cells in an *in vitro* specific antibody response. *Eur. J. Immunol.*, **12**, 232.

EPSTEIN, M.A. '1978). Epstein–Barr virus discovery, properties and relationship to nasopharyngeal carcinoma. W. Davies (ed). *Nasopharyngeal Carcinoma: Aetiology and Control.* (Ed. de-The *et al.*) Lyon: IARC p. 333.

EPSTEIN, M.A. & ACHONG, B.G. (1979). The relationship of the virus to Burkitts lymphoma. *The Epstein–Barr Virus.* (Eds. Epstein & Achong), Berlin: Springer-Verlag.

EVANS, K.L., FALDETTA, T.J., HUMPHREYS, R.E., PRATT, D.W., YURIS, E.J. & SCHLOSSMAN, S.F. (1978). Peripheral human T cells sensitised in mixed leucocyte culture synthesize and express Ia-like antigens. *J. Exp. Med.*, **148**, 1440.

EVANS, A.S., NIEDERMAN, J.C. & McCOLLUM, R.W. (1968). Seroepidemiologic studies of infectious mononucleosis with EB virus. *N. Engl. J. Med.*, **279**, 1121.

GOLDEN, H.D., CHANG, R.S., PRESCOTT, W., SIMPSON, E., & COOPER, T.Y. (1973). Leucocyte-transforming agent: prolonged excretion by patients with mononucleosis and excretion by normal individuals. *J. Infect. Dis.*, **127**, 471.

HEWETSON, J.F., ROCCHI, G., HENLE, W. & HENLE, G. (1973). Neutralizing antibodies to Epstein-Barr virus in healthy populations and patients with infectious mononucleosis. *J. Inf. Dis.*, **128**, 283.

HENLE, G., HENLE, W. & DIEHL, V. (1968). Relation of Burkitts tumour associated herpes-type virus to infectious mononucleosis. *Proc. Natl Acad. Sci.*, **59**, 94.

KAPLAN, M.E. & CLARK, C. (1974). An improved rosetting assay for detection of human T lymphocytes. *J. Immunol. Methods*, **5**, 131.

MILLER, G., SHOPE, T., LISCO, H., STITT, D. & LIPMAN, M. (1972). Epstein–Barr virus: Transformation cytopathic changes and viral antigens in squirel monkey and marmoset leucocytes. *Proc. Natl Acad. Sci.*, **69**, 383.

MISKO, I.S., KANE, R.G & POPE, J.H. (1982). Generation *in vitro* of HLA-restricted EB virus specific cytotoxic human T cells by autologous lymphoblastoid cell lines: the roles of previous EB virus infection and foetal calf serum. *Int. J. Cancer*, **29**, 41.

MOSS, D.J., RICKINSON, A.B. & POPE, J.H. (1978). Long term T cell mediated immunity to Epstein–Barr virus in man. I. Complete regression of virus-induced transformation in cultures of seropositive donor leucocytes. *Int. J. Cancer*, **22**, 662.

MOSS, D.J., WALLACE, L.E., RICKINSON, A.B. & EPSTEIN, M.A. (1981). Cytotoxic T cell recognition of Epstein–Barr virus infected B cells. I. specificity and HLA restriction of effector cells reactivated *in vitro*. *Eur. J. Immunol.*, **11**, 686.

NILSSON, K., KLEIN, G., HENLE, W. & HENLE, G. (1971). The establishment of lymphoblastoid lines from adult and foetal human lymphoid cells and its dependence on EBV. *Int. J. Cancer*, **8**, 443.

PALACIOS, R. & MOLLER, G. (1981). Cyclosporin A blocks receptors for HLA-DR antigens on T cells. *Nature*, **290**, 792.

PEREIRA, R.S., DORE, C.G. & GEAR, A.J. (1982). Rheumatoid lymphocyte defect revealed by cyclosporin A. *Lancet*, **ii**, 721.

RICKINSON, A.B., MOSS, D.J., POPE, J.H. (1979). Long-term T cell mediated immunity to Epstein–Barr virus in man. II. Components necessary for regression in virus-infected leucocyte cultures. *Int. J. Cqncer*, **23**, 610.

RICKINSON, A.B., WALLACE, L.E. & EPSTEIN, M.A. (1980). HLA-restricted T cell recognition of Epstein–Barr virus infected B cells. *Nature*, **283**, 865.

THOMAS, Y., SOSMAN, J., ROGOZINSKI, L. & 4 others. (1981). Functional analysis of human T cell subsets defined by monoclonal antibodies. III. Regulation of helper factor production by T cell subsets. *J. Immunol.*, **126**, 1948.

THORLEY-LAWSON, D.A. (1981). The transformation of adult—but not newborn human lymphocytes by Epstein–Barr virus and phytohaemagglutinin is inhibited by interferon. The early suppression of Epstein–Barr virus infection is mediated by interferon. *J. Immunol.*, **126**, 829.

TSOUKAS, C.D., FOX, R.I., SLOVIN, S.F. & 4 others. (1981). T lymphocyte-mediated cytotoxicity against autologous EBV-genome bearing B cells. *J. Immunol.*, **126**, 1742.

YAN, B., BEVERLEY, P.C.L., KNOWLES, R.W. & BODMER, W.F. (1983). Two monoclonal antibodies identifying a subset of human peripheral mononuclear cells with NK and K cell activity. *Eur. J. Immunol.* (in press).